# A new mouse model of frailty: the Cu/Zn superoxide dismutase knockout mouse

**DOI:** 10.1007/s11357-017-9975-9

**Published:** 2017-04-13

**Authors:** Sathyaseelan S. Deepa, Shylesh Bhaskaran, Sara Espinoza, Susan V. Brooks, Anne McArdle, Malcolm J. Jackson, Holly Van Remmen, Arlan Richardson

**Affiliations:** 1 Department of Geriatric Medicine and the Reynolds Oklahoma Center on Aging, Oklahoma University Health Science Center, Oklahoma City, OK USA; 2 Aging and Metabolism Research Program, Oklahoma Medical Research Foundation, Oklahoma City, OK USA; 3 Barshop Institute for Longevity & Aging Studies, Medicine, Division of Geriatrics, Gerontology & Palliative Medicine, University of Texas Health Science Center at San Antonio, San Antonio, TX USA; 4 Geriatrics Research, Education & Clinical Center, South Texas Veterans Health Care System, San Antonio, TX USA; 5 Department of Molecular and Integrative Physiology, Institute of Gerontology, University of Michigan, Ann Arbor, MI USA; 6 Department of Musculoskeletal Biology, MRC Arthritis Research UK Centre for Integrated Research into Musculoskeletal Ageing (CIMA), Institute of Ageing and Chronic Disease, University of Liverpool, Liverpool, UK; 7 Oklahoma City VA Medical Center, Oklahoma City, OK USA

**Keywords:** Cu/Zn superoxide dismutase, Frailty, Inflammation, Senescence, Sarcopenia, Oxidative stress

## Abstract

Frailty is a geriatric syndrome that is an important public health problem for the older adults living in the USA. Although several methods have been developed to measure frailty in humans, we have very little understanding of its etiology. Because the molecular basis of frailty is poorly understood, mouse models would be of great value in determining which pathways contribute to the development of frailty. More importantly, mouse models would be critical in testing potential therapies to treat and possibly prevent frailty. In this article, we present data showing that Sod1KO mice, which lack the antioxidant enzyme, Cu/Zn superoxide dismutase, are an excellent model of frailty, and we compare the Sod1KO mice to the only other mouse model of frailty, mice with the deletion of the *IL-10* gene. Sod1KO mice exhibit four characteristics that have been used to define human frailty: weight loss, weakness, low physical activity, and exhaustion. In addition, Sod1KO mice show increased inflammation and sarcopenia, which are strongly associated with human frailty. The Sod1KO mice also show alterations in pathways that have been proposed to play a role in the etiology of frailty: oxidative stress, mitochondrial dysfunction, and cell senescence. Using Sod1KO mice, we show that dietary restriction can delay/prevent characteristics of frailty in mice.

## Introduction

Frailty is a clinical geriatric syndrome that is an important public health problem affecting a large proportion of older adults in the USA. Approximately 10% of older adults are frail; however, this prevalence increases markedly to 30% in those over 80 years of age (Collard et al. 2012; Mohler et al. 2014). The cost of frailty, including costs from falls and disability, was estimated to be more than $18 billion in 2000 (Janssen et al. 2004). This syndrome is recognized by geriatricians as a syndrome in which individuals show a progressive physical decline that is associated with increased medical comorbidity, disability, and mortality (Fried et al. 2001; Woods et al. 2005) even after taking common age-associated diseases and conditions into consideration (Fried and Walston 1998). As shown in Table 1, Fried et al. (2001) operationally defined and validated frailty as a medical syndrome of weight loss, weakness, slowness in walking, low physical activity, and exhaustion. Individuals with three or more of these five characteristics are categorized as frail, individuals with one or two are categorized as pre-frail, and individuals with none are classified as non-frail. These criteria were modeled in the Cardiovascular Health Study. In this cohort, individuals identified as frail were at significantly increased risk of hospitalization, falling, worsening disability, and death, after adjustment for multiple potential confounding factors, e.g., socioeconomic status, health status, subclinical and clinical disease, depressive symptoms, and disability status at baseline (Fried et al. 2001). In addition, the Cardiovascular Health Study showed that the increased risk for adverse health outcomes followed a stepwise pattern of increasing risk by frailty categorization, i.e., pre-frail conferred higher risk than non-frail and frail conferred higher risk than pre-frail, suggesting a dose–response relationship. In 2004, Studenski et al. developed a Clinical Global Impression of Change in Physical Frailty (CGIC-PF) instrument to assess physical frailty. As shown in Table 1, the CGIC-PF included six intrinsic domains (mobility, balance, strength, endurance, nutrition, and neuromotor performance) and seven consequence domains (medical complexity, healthcare utilization, appearance, self-perceived health, activities of daily living, emotional status, and social status) with each domain containing two to four clinical indicators. Changes were scored on a 7-point scale from markedly worse to markedly improved. Jones et al. (2004) developed a frailty screening tool based on items obtained from a comprehensive geriatric assessment, including impairments, disability, medical comorbidity, and quality of life. Rockwood et al. (2005) subsequently developed the 7-point clinical frailty scale in order to provide a simpler, more clinically useful tool for frailty screening. Therefore, while there is no clear consensus for frailty screening in the clinical setting, the frailty phenotype proposed by Fried et al. (2001) is the most extensively studied and has been cross-validated in several other cohorts, including the Women’s Health and Aging Studies (Bandeen-Roche et al. 2006), the Women’s Health Initiative (Woods et al. 2005), and the San Antonio Longitudinal Study of Aging (Espinoza and Hazuda 2008). One of the hallmark physical characteristics of frailty is sarcopenia, loss of muscle mass, and function. Sarcopenia is considered as a major underlying component of frailty and was included in conceptual framework, originally put forth by Fried and Walston (2003). Although the causal relationship between sarcopenia and frailty is unclear, Landi et al. (2015) argue that sarcopenia is a biological substrate of physical frailty.

**Table 1:** The frailty phenotype criteria and measurements in humans

Fried et al. (2001)	Studenski et al. (2004)
1. Shrinking Weight loss (>10 lbs. lost unintentionally in prior year), sarcopenia (loss of muscle mass) 2. Weakness Grip strength: lowest 20% (by gender, body mass index) 3. Poor endurance and energy Self-reported exhaustion 4. Slowness Walking time/15 ft: slowest 20% (by gender, height) 5. Low physical activity level Kcals/week: lowest 20% (by gender)	1. Strength (grip, upper, lower) 2. Balance (falls, fear of falling, balance performance) 3. Motor processing (coordination, movement planning, and speed) 4. Flexibility (stiffness, range of motion) 5. Nutrition (intake, weight, muscle mass, synthetic function) 6. Stamina (energy/fatigue, endurance) 7. Mobility (walking, transfers, stairs, devices) 8. Vulnerability (utilization, ability to tolerate stress) 9. Neurocognitive processing (alertness, attention, multi-tasking) 10. Self-perceived health 11. Life space (ability to leave room, home, neighborhood, community) 12. Activities of daily living (basic, instrumental, advanced) 13. Modifications (devices, frequency, or way of doing) 14. Role function (social, vocational) 15. Interest and motivation (interest in others, desire to take on activities) 16. Self-efficacy (confidence, locus of control) 17. Emotions (depression, anxiety) 18. Appearance (interest and awareness of grooming) 19. Behavior/personality (self vs. other orientation, irritability) 20. Social world (interest and interactions with others)

Frailty is thought to represent a distinct physiologic state in which individuals are highly vulnerable to external stressors and recover poorly from these, rarely reaching their previous baseline of health and function (Fried and Walston 2003), i.e., it is characterized by loss of resilience, increased vulnerability to stress, and a lack of physiological reserve. Currently, a large amount of evidence suggests that low-grade inflammation plays an important role in the etiology of frailty. The initial studies showing an association between frailty and inflammation were obtained with serum or plasma markers of inflammation, such as interleukin-6 (IL-6) and C-reactive protein (CRP). Walston et al. (2002) found in the Cardiovascular Health Study that frail individuals had increased mean levels of CRP compared to non-frail individuals (5.5 ± 9.8 vs. 2.7 ± 4.0 mg/L). These peripheral blood markers persisted after exclusion of individuals with cardiovascular disease and diabetes, as well as after adjustment for age, sex, and race. Subsequently, Leng et al. (2002) found in community-dwelling older adults that serum IL-6 levels were elevated over 50% in frail compared to non-frail older adults. These findings have been replicated by Barzilay et al. (2007). Qu et al. (2009) found that expression of a potent inflammatory chemokine, CXCL-10, by monocytes was increased in frail compared to non-frail elderly, and there was high correlation between monocyte CXCL-10 expression and serum IL-6 levels. In addition, they found that monocytes from frail individuals had twofold increased expression of inflammatory genes compared to non-frail individuals. More recently, Collerton et al. (2012) showed an association of IL-6, TNF-α, and CRP levels with frailty in a large cross-sectional cohort in Scotland that included 845 subjects >85 years of age.

It has been argued that the increased inflammation observed in frail older adults might arise from increased oxidative stress. Oxidative stress increases with age and multiple studies report an association between oxidative stress and frailty. Protein carbonyls, which are a result of oxidative damage to proteins, were shown in a cross-sectional study to be associated with poor handgrip strength (Howard et al. 2007), and serum protein carbonyl levels were found to be an independent predictor of gait speed decline in humans (Semba et al. 2007). High levels of isoprostanes, which are produced by lipid peroxidation, have been reported to predict death in older adults (Cesari et al. 2012). In 2009, two groups reported that high oxidative stress was correlated with frailty. Serviddio et al. (2009) found increased levels of oxidized glutathione and protein adducts of malonaldehyde and 4-hydroxy-2,3-nonenal in the plasma of frail patients. Wu et al. (2009) showed an association between a marker of oxidative DNA damage (8-oxo-deoxyguanosine) in the plasma and frailty in elderly Chinese. More recently, Ingles et al. (2014) reported that markers of oxidative stress in plasma (malonaldehyde and protein carbonylation) were related to frailty but not to age or sex in a geriatric population, and in the Framingham Offspring Study, Liu et al. (2016) reported that elevated levels of plasma isoprostanes were associated with increased risk of frailty and slower gait speed.

Mitochondrial dysfunction has been proposed to play a role in frailty because mitochondria are the major source of reactive oxygen species (ROS) and energy in the cell. Therefore, changes in the function of mitochondria leading to increased ROS generation or reduced ATP generation could lead to frailty. For example, the increased oxidative stress that has been observed in frail older adults has been hypothesized to arise from mitochondrial dysfunction (Villamena and Zweier 2004). Consistent with this hypothesis is the study by Baptista et al. (2012) showing that superoxide anion production was increased in white blood cells of frail older adults. Recently, it was also shown in blood that, lower mtDNA copy number, a marker that reveals mitochondrial depletion, energy reserves, and oxidative stress is associated with frailty (Ashar et al. 2015).

More recently, it has been proposed that cell senescence may play a role in frailty (Fedarko 2011; LeBrasseur et al. 2015). It is now well accepted that senescent cells increase with age in many mammalian tissues and are found at sites of age-related pathologies (for reviews see Coppé et al. 2010 and Rodier and Campisi 2011). In addition, Campisi’s laboratory has shown that senescent cells secrete biologically active proteins (e.g., growth factors, proteases, cytokines, and other factors) that have potent autocrine and paracrine activities (Coppe et al. 2008), a phenomenon termed the senescence-associated secretory phenotype (SASP). The SASP includes several potent inflammatory cytokines including IL-6, IL-1β, and IL-8, which may serve as an important source of low-level chronic inflammation (Chung et al. 2009). Therefore, it is possible that increased cell senescence in frail older adults might contribute to increased chronic inflammation and the frailty phenotype. Baker et al. (2011) published the first direct evidence showing that cell senescence is involved in aging in mice. Using a mouse model they developed in which senescent, p16^Ink4a^-positive cells were eliminated upon the administration of the drug, AP20187, they were able to test the effect of reducing/eliminating senescent cells on aging/pathology. AP20187 treatment delayed/reduced the development of age-associated deficits known to accompany cell senescence, e.g., sarcopenia and the loss of adipose tissue. Of particular importance to frailty was the effect of eliminating senescent cells on muscle. Muscle fiber diameters of the AP20187-treated mice were larger than those of untreated mice and treadmill exercise tests revealed that duration of exercise, distance traveled, and overall amount of work performed were all significantly increased in the mice treated with AP20187, indicating preservation of muscle function by the elimination of senescent cells. They also showed that improvements in skeletal muscle function could also be achieved by the clearance of p16^Ink4a^-positive senescent cells.

## Animal models of frailty

Over the past two decades, the use of genetically modified mouse models to study human diseases has become very popular, e.g., the use of ApoE or LDL receptor knockout mice to model atherosclerosis, which does not occur in normal mice, or the use of transgenic mice that produce a mutant form of a gene that has been shown to be associated with a human disease, e.g., transgenic mice that overexpress mutated amyloid precursor protein (Alzheimer’s disease), mutant Cu/Zn superoxide dismutase (amyotrophic lateral sclerosis), or mutant huntingtin (Huntington’s disease). The major advantage of mouse models that mimic a human disease is that one can use mice to identify potential targets for developing therapies as well as the biological mechanism(s) underlying the disease to be defined.

Because the molecular basis of frailty, a syndrome rather than a disease, is poorly understood, mouse models of frailty would be of great value in determining what pathways contribute to the development of frailty. More importantly, a mouse model of frailty would give the geriatric research community a model to use in testing potential therapies to treat and possibly prevent frailty. This is particularly important in treating frailty because older, frail individuals are in poor health and have limited resilience capacity.

### Interleukin-10 knockout mouse

Interleukin-10 (IL-10) was identified as a cytokine produced by type 2 T helper cells in 1990 (Moore et al. 1990), and IL-10^−/−^ (IL-10KO) mice have been generated to study the physiological role of IL-10. Kuhn et al. (1993) showed that IL-10KO mice develop a chronic enterocolitis because of an aberrant immune response. Subsequently, it was found that knocking out IL-10 resulted in an increased activation of nuclear factor-kappa B (NFκB) induced inflammatory mediators (Rennick et al. 1995; Berg et al. 1996). Because IL-10KO mice showed increased inflammation and because inflammation was believed to play a major role in frailty, Walston et al. (2008) determined whether the IL-10KO mice exhibited a frailty phenotype. As would be expected, the IL-10KO mice showed increased serum levels of IL-6. Studying mice from 10 to 18 months of age, Walston et al. (2008) found that the IL-10KO mice showed a greater decrease in grip strength with increasing age than wild-type (WT) mice and 125 genes were differentially expressed in the skeletal muscle of 50-week-old IL-10KO and WT mice. No major differences in body weight or measures of activity were found. Walston’s group (Ko et al. 2012) subsequently compared IL-10KO and WT mice from 12 to 90 weeks of age and found that the levels of circulating pro-inflammatory cytokines, e.g., IFN-γ, IL-1β, IL-6, KC, and TNF-α, were elevated across age groups in the IL-10KO mice. They also found a significant difference in the mortality between the IL-10KO and WT mice; the median survival of the IL-10KO mice was ~21 months while less than 10% of the WT mice had died at this age. IL-10KO mice also show mitochondrial dysfunction as suggested by lower rates of ATP synthesis and higher levels of damaged mitochondria in skeletal muscle (Akki et al. 2014; Ko et al. 2016).

### Cu/Zn superoxide dismutase (Sod1KO) knockout mouse

Cu/Zn superoxide dismutase (Cu/ZnSOD) is the major superoxide dismutase isozyme that catalyzes the conversion of superoxide anions to hydrogen peroxide. It is found in all cells and is localized in the cytosol and the intermembrane space of the mitochondria (Okado-Matsumoto and Fridovich 2001). In 1996, Reaume et al. generated mice null for CuZnSOD (Sod1KO) and reported that they appear normal at birth. However, Sod1KO females are almost totally infertile due to ovarian dysfunction (Ho et al. 1998; Matzuk et al. 1998), and the Sod1KO mice show very high levels of oxidative stress in various tissues and plasma (Muller et al. 2006). In 2005, Huang’s laboratory reported that Sod1KO mice show a decrease in lifespan of approximately 30% (Elchuri et al. 2005). In 2013, our group confirmed that the lifespan of Sod1KO mice was reduced, e.g., median lifespan was reduced ~25% from ~30 months for WT mice to ~22 months for Sod1KO mice (Zhang et al. 2013a). Because Elchuri et al. (2005) reported that more than 70% of the Sod1KO mice developed liver hyperplasia and hepatocellular carcinoma later in life, it was initially believed that the decrease in the lifespan of the Sod1KO mice was the result of the dramatic increase in hepatocellular carcinoma, which is rare in C57BL/6 mice. In a more recent study, we found that only about 30% of the Sod1KO mice developed hepatocellular carcinoma later in life (Zhang et al. 2013a). However, data from several studies show that Sod1KO mice exhibited various accelerated aging phenotypes [e.g., hearing loss (Keithley et al. 2005), cataracts (Olofsson et al. 2007), skin thinning and delayed wound healing (Iuchi et al. 2010), and loss of muscle mass (Muller et al. 2006)] suggesting that the reduced lifespan of Sod1KO mice is largely a result of accelerated aging. We also showed that dietary restriction, which is a manipulation that retards aging in rodents, increased the lifespan of the Sod1KO mice to that of normal WT mice (Zhang et al. 2013a). Thus, the current data indicate that the Sod1KO mice show an accelerated aging phenotype.

**Fig. 1:**
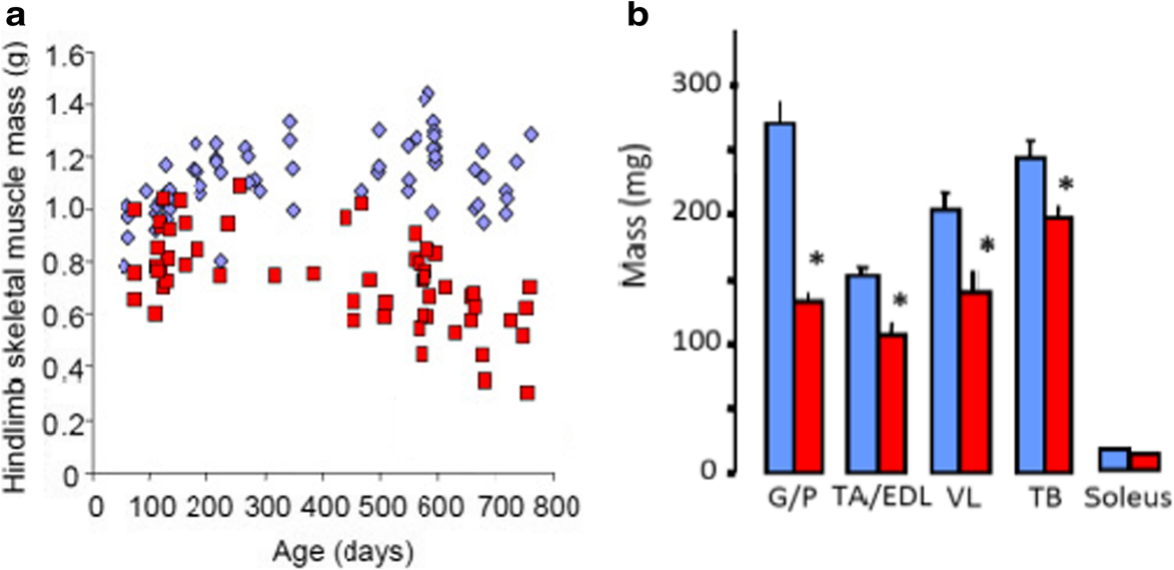
Muscle mass is reduced in Sod1KO mice. **a** Hindlimb muscle mass of WT and Sod1KO mice over the lifespan of the Sod1KO mice. The age-dependent decrease in muscle mass in the Sod1KO mice, either absolute or relative to body mass, was statistically significant (*P* < 0.05) as determined by general linear model ANOVA. **b** Mass in milligrams for individual muscles from 20-month-old WT and Sod1KO mice. *G/P* gastrocnemius and plantaris, *TA/EDL* tibialis anterior and extensor digitorum longus, *VL* vastus lateralis, *TB* triceps brachii. *Blue squares and bars* represent WT and *red squares and bars* represent Sod1KO. Data were analyzed by general linear model ANOVA *P* < 0.05) with Tukey’s post hoc indicated by an *asterisk*. Data taken from Muller et al. (2006)

**Fig. 2:**
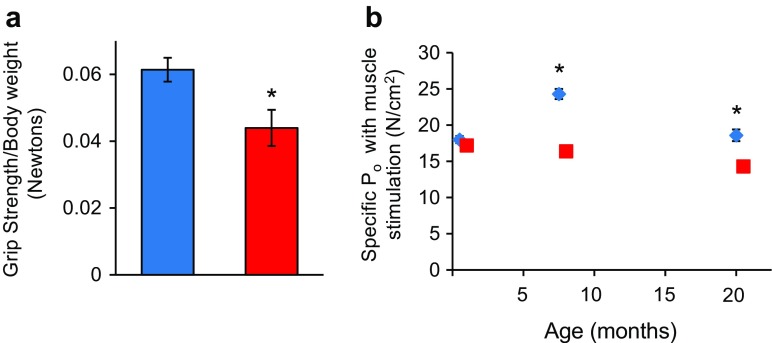
Muscle strength is reduced in Sod1KO mice. **a** Grip strength of 9-month-old male WT (*n* = 7) and Sod1KO mice (*n* = 8). Data are expressed as mean ± SEM. Data were analyzed by unpaired *t* test (*P < 0.05). **b** Maximum isometric specific force (specific Po) values for gastrocnemius muscle of WT and Sod1KO mice during direct muscle stimulation. Data for 1-, 8-, and 20-month-old mice were compared by two-factor (genotype × age) ANOVA. When the ANOVA showed significant differences between groups, individual differences were established by Bonferroni post hoc analyses. Significance was set at *P <* 0.05 (data taken from Larkin et al. 2011). *Blue squares and bars* represent WT and *red squares and bars* represent Sod1KO

We began considering that Sod1KO mice might be a mouse model of frailty because they exhibited many of the physiological deficits that characterize frailty in humans, e.g., weight loss, weakness, low physical activity, and exhaustion which were identified by Fried et al. (2001). Although the Sod1KO mice and WT mice are initially similar sizes, the growth of the Sod1KO mice is reduced resulting in ~20% decrease in body mass of the Sod1KO mice compared to WT mice (Muller et al. 2006). More important, as shown in Fig. 1a, the Sod1KO mice have an accelerated loss in muscle mass after 6 months of age compared to WT mice. Figure 1b shows that a significant decrease in the mass of all muscle types is observed in *Sod1*KO mice except for soleus. The greatest decrease in mass is observed in the gastrocnemius, which is the muscle most affected by aging in WT mice. The Sod1KO mice also show a significant decrease (~30%) in muscle strength as measured by a reduced grip strength (Fig. 2a). In addition, Larkin et al. (2011) showed that the strength of the muscle from Sod1KO mice was reduced. The lower force generating capacity was not explained entirely by the smaller muscle mass because when the force was normalized by muscle fiber cross-sectional area, the force generating capacity of the gastrocnemius muscle from the Sod1KO mice was still ~45% lower than WT mice indicating a decrease in the quality as well as in the quantity of muscle (Fig. 2b). Thus, the Sod1KO mice show marked increase in sarcopenia, which is a hallmark of frailty in humans.

**Fig. 3:**
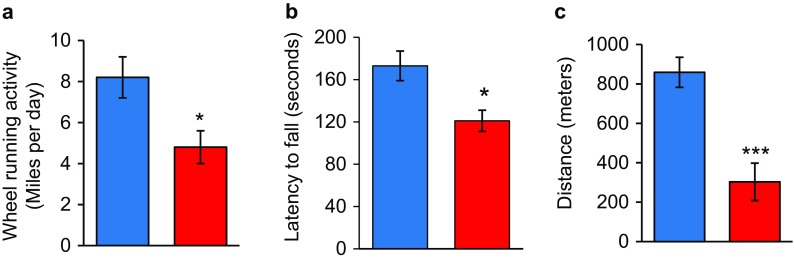
Voluntary wheel running, rotarod performance, and treadmill endurance are reduced in Sod1KO mice. **a** Voluntary wheel running. WT and Sod1KO mice were individually housed in cages equipped with a zero-resistance running wheel and the average distance run each by each mouse over 16 weeks is shown. *P < 0.05 by ANOVA (data taken from Muller et al. 2006). **b** Rotarod performance. The average time in seconds that the mice remain on the rotating rod before falling off. *P < 0.05 by ANOVA (data taken from Muller et al. 2006). **c** Treadmill endurance. The time to run to exhaustion on a treadmill is shown for WT and Sod1KO mice. Significance was established using Student’s *t* test, ***P < 0.001 (data taken from Jang et al. 2010). *Blue bars* represent WT and *red bars* represent Sod1KO

**Fig. 4:**
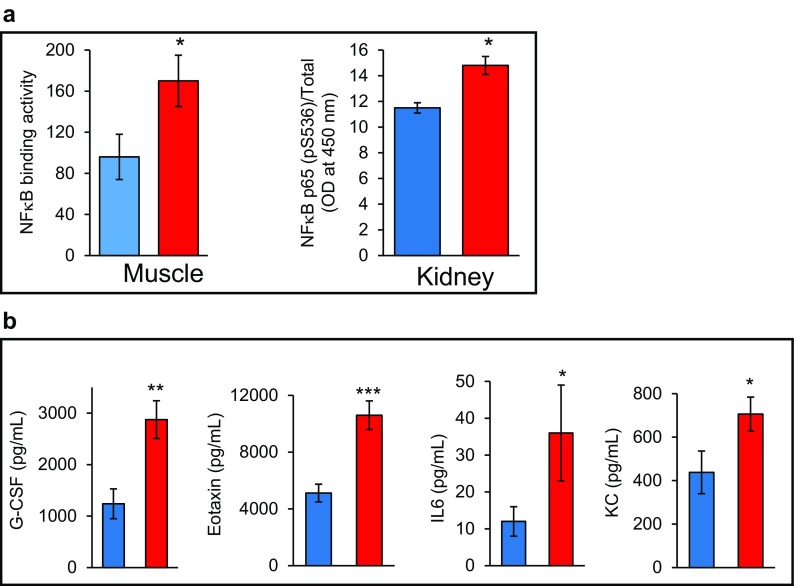
Measures of inflammation are increased in Sod1KO mice. **a** NFκB binding activity of nuclear extracts from gastrocnemius muscle data was analyzed using ANOVA (data taken from Vasilaki et al. 2010) and kidney analyzed by two-tailed Student’s *t* test (data are taken from Zhang et al. (2017)) of WT and Sod1KO mice, *P < 0.05. **b** The levels of cytokines were measured in the serum collected from WT and Sod1KO mice and data were analyzed by one-tailed Student’s *t* test, *P < 0.05, **P < 0.01, ***P < 0.001 (data taken from Zhang et al. 2017). *Blue bars* represent WT and *red bars* represent Sod1KO

**Fig. 5:**
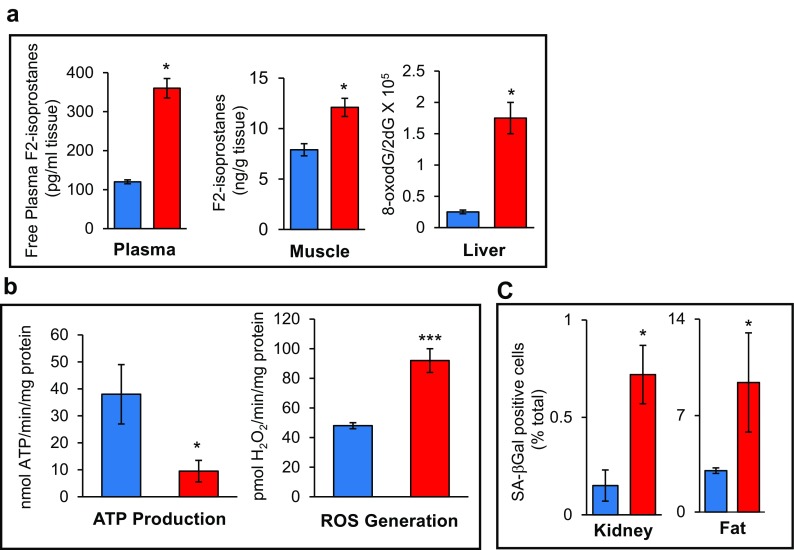
Oxidative stress, mitochondrial dysfunction, and cell senescence are increased in Sod1KO mice. **a** Oxidative damage. The level of lipid peroxidation (F_2_-isoprostanes) in plasma and whole hind limb skeletal muscle isoprostane. Data were analyzed using ANOVA and Tukey’s post hoc test, *P < 0.05) (data taken from Muller et al. 2006) and DNA oxidation (8-oxo-deoxyguanosine) in liver analyzed by the non-parametric test of ANOVA. Values that are significantly different (*P* < 0.05) (data taken from Perez VI et al. 2009) from WT and Sod1KO mice. **b** Mitochondrial dysfunction. The rate of ATP generation and hydrogen peroxide generation by isolated mitochondria from 20-month-old wild-type and Sod1KO mice is shown and the data were analyzed by Student’s *t* test, *P < 0.05, **P < 0.001 (data taken from Jang et al. 2010). **c** Cell senescence. The percent β-gal staining cells in kidney (data taken from Zhang et al. 2017) and fat tissue from Sod1KO and WT mice. Data were analyzed by Student’s *t* test, *P < 0.05. *Blue bars* represent WT and *red bars* represent Sod1KO

In addition to the loss of muscle mass and weakness, which are hallmarks of frailty, the Sod1KO mice also exhibit other phenotypes that are observed in humans, e.g., reduced physical activity and reduced endurance. The Sod1KO mice do not show any change in cage activity; however, spontaneous wheel running activity of the Sod1KO mice is dramatically reduced compared to the WT mice as shown in Fig. 3a (Muller et al. 2006). Although there are no data on walking speed of the Sod1KO mice, rotarod performance has been measured, which is a measure of endurance and muscle strength and has been used as a surrogate of maximal walking speed in mice (Liu et al. 2014). As shown in Fig. 3b, Sod1KO mice also show ~30% decrease in rotarod performance (Muller et al. 2006). Reduced endurance and exhaustion is also a phenotype of frailty in humans. We also measured the endurance exercise capacity of Sod1KO mice using treadmill running to exhaustion (Jang et al. 2010). Figure 3c shows that the distance that Sod1KO mice run to exhaustion is only about one third of the distance the WT mice run (e.g., 303 ± 95 vs. 859 ± 76 m). We also found that the Sod1KO mouse shows changes in pathways/processes that have been proposed to play a role in the etiology of frailty. As noted above, an increase in low-grade inflammation is believed to play an important role in frailty. We have several pieces of data indicating that inflammation is increased in the Sod1KO mice. First, we have found that the NFκB activation, which is involved in the transcription of inflammatory genes, is increased in skeletal muscle and kidney of Sod1KO mice compared to WT mice (Fig. 4a). Second, we measured a panel of pro-inflammatory cytokines in the sera of Sod1KO and WT mice (Zhang et al. 2017). As shown in Fig. 4b, four of the ten cytokines measured were significantly increased in the Sod1KO mice. If we combined the total content of the ten cytokines in the sera, we observed over a 1.8-fold increase in the level of circulating pro-inflammatory cytokines in the Sod1KO mice. Increased oxidative stress has also been correlated with frailty in humans (Serviddio et al. 2009; Wu et al. 2009; Ingles et al. 2014). As would be expected, Sod1KO mice are characterized by high levels of oxidative damage (Perez et al. 2009; Muller et al. 2006). As shown in Fig. 5a, we observed increased lipid peroxidation in plasma and muscle and increased oxidative damage to DNA in liver of the Sod1KO mice. Mitochondrial dysfunction, which has also been proposed to play a role in frailty (Walston et al. 2006; Wu et al. 2014), is also observed in Sod1KO mice. As shown in Fig. 5b, mitochondria from skeletal muscle of Sod1KO mice show increased production of reactive oxygen species and reduced ATP production. More recently, it has been argued that the accumulation of senescent cells could be involved in frailty (Tchkonia et al. 2013; Mohler et al. 2014), and as shown in Fig. 5c, we have recently found that cell senescence is increased in kidney and fat tissue of Sod1KO mice compared to WT mice.

## Discussion

Table 2 compares the phenotypes of frailty in humans to IL-10KO and Sod1KO mice. Both the IL-10KO and Sod1KO mice exhibit a significant decrease in lifespan, suggesting that they might physiologically age more rapidly than WT mice and therefore would be more prone to developing sarcopenia at a younger age. The IL-10KO mice, which were originally proposed by Walston et al. (2008) as a model of frailty, exhibit increased inflammation and reduced grip strength; however, there is limited information on whether the IL-10KO mice exhibit other characteristics of human frailty. In contrast, a great deal of information has been generated over the past decade on the Sod1KO mice, which indicates that Sod1KO mice would be a good model of frailty. As can be seen from Table 2, the Sod1KO mice exhibit changes in four of the five defining characteristics of human frailty proposed by Fried et al. (2001): weight loss, weakness, low physical activity, and exhaustion. In addition, the Sod1KO mice show increased circulating levels of pro-inflammatory cytokines and develop sarcopenia, which are strongly associated with frailty in humans. The Sod1KO mice also show alterations in pathways that have been proposed to play a role in the etiology of frailty: oxidative stress, mitochondrial dysfunction, and cell senescence.

**Table 2:** Comparison of frailty phenotypes in humans and mouse models of frailty

Frailty phenotypes	Humans	IL-10KO mice	Sod1KO mice
Body weight	Reduced	No change	Reduced
Muscle mass	Reduced	Unknown	Reduced
Weakness	Grip strength reduced	Grip strength reduced	Grip and muscle strength reduced
Walking speed	Reduced	Unknown	Unknown
Physical activity	Low	No change in spontaneous cage activity	Reduced voluntary running wheel activity
Endurance/exhaustion	Reduced	Unknown	Treadmill and rotarod performance reduced
Inflammation	Increased levels of IL-6, CRP, CXCL-10, and TNF-α	Increased levels of IL-6, IFN-γ, IL-1β, KC, and TNF-α	Increased levels of IL-6, GM-CSF, Eotaxin, and KC
Lifespan	Reduced	Reduced	Reduced

Although the IL-10KO and Sod1KO mouse models are currently the best mouse models of frailty available, there is always the question of how accurately deleting *IL-10* or *Sod1* replicates frailty because there is no evidence that the expression of either of these genes plays a role in frailty in humans. In addition, these mice develop special pathologies normally not observed in the WT mice, e.g., chronic enterocolitis in the IL-10KO mice and hepatocellular carcinoma in about one third of the Sod1KO mice. Nevertheless, the IL-10KO and Sod1KO mouse models give investigators the first tools for studying the mechanism underlying frailty and testing interventions to delay/prevent frailty. Important information about frailty has already been gained from these models. For example, they have provided direct evidence for inflammation playing an important role in frailty. The data by Walston et al. (2008) with IL-10KO mice in which inflammation is induced demonstrates that inflammation most likely plays a causative role in frailty. Our data showing that Sod1KO mice have increased inflammation also support the concept that inflammation plays a role in the etiology of frailty. In addition, the Sod1KO mouse model provides our first insight into how frailty might be prevented/delayed. Frailty in Sod1KO mice is attenuated by dietary restriction, e.g., dietary restriction reversed the loss of muscle mass and function and improved mitochondria function (Jang et al. 2012) and attenuated the increase in oxidative damage, cell senescence, and circulating levels of IL-6 (Zhang et al. 2017). These data are supported by a recent study from Kane et al. (2016) showing that 6 months of dietary restriction improved the frailty index score of 24-month-old WT mice.

In summary, the IL-10KO and Sod1KO mouse models give investigators the first animal models that can be used to study frailty. These mouse models allow investigators to use genetic approaches to identify the pathways that can lead to frailty as well as determine which tissues are key in the development of frailty. Because these two models induce frailty through different pathways (e.g., immune dysfunction for the IL-10KO mice and increased oxidative stress in the Sod1KO mice), they are complementary and a combination of these models will give investigators a robust approach to identifying the biological processes underlying frailty. In addition, the use of conditional Sod1KO models allow the investigators to study the effect of the tissue-specific deletion of Cu/Zn superoxide dismutase on frailty. Sod1^flox/flox^ have been generated (Zhang et al. 2013b) and used to study the role of the muscle and motor neurons in sarcopenia (Zhang et al. 2013b; Sataranatarajan et al. 2015).
